# Professional Values of Nurses and Nursing Students: a comparative study

**DOI:** 10.1186/s12909-019-1878-2

**Published:** 2019-11-27

**Authors:** Batool Poorchangizi, Fariba Borhani, Abbas Abbaszadeh, Moghaddameh Mirzaee, Jamileh Farokhzadian

**Affiliations:** 1 Department of ICU, Shahid Bahonar Hospital, Kerman University of Medical Sciences, Kerman, Iran; 2 Department of Nursing Ethics, Medical Ethics and Law Research Center, Shahid Beheshti University of Medical Sciences, Tehran, Iran; 3 Bam University of Medical Sciences, Bam, Iran; 4 Department of Biostatistics and Epidemiology, School of Public Health, Kerman University of Medical Sciences, Kerman, Iran; 5 Nursing Research Center, Kerman University of Medical Sciences, PO Box: 7716913555, Kerman, Iran

**Keywords:** Values, Professional values, Nursing education, Nurses, Nursing students

## Abstract

**Background:**

The process of learning the professional values is started from the student’s entering to the university and to the workplace. This study compared the importance of professional values from the perspectives of nurses and nursing students.

**Methods:**

This descriptive-analytical study was conducted on 250 nurses and 100 nursing students. Data were collected using the Nursing Professional Values Scale-Revised.

**Results:**

According to the findings, the mean scores of nurses (3.68 ± 0.16) and nursing students’ (3.86 ± 0.17) perspective toward professional values were at an important level. Furthermore, the students’ perspective toward the professional values’ importance was significantly more favorable than those of nurses. The highest mean scores of professional values in the two groups were related to the caring and justice domains. Both groups considered activism and professionalism as the least important domains among the others.

**Conclusions:**

As the findings suggest, we need to pay more attention to values training, especially professionalism and activism, during undergraduate education for preparing the nurses to work in today’s complex healthcare context. It is necessary to conduct more comprehensive studies for exploring the gap between theory and practice in different cultures and contexts.

## Background

Health care professions, especially nursing, have outstanding and distinctive values as dynamic phenomena [1]. Professional values are associated with individuals’ beliefs, as members of a profession, about the appropriateness and desirability of something [2]. Moreover, values are crucial factors of what motivates and rewards nurses [3]. Professional values often lead to development of the personal values [4]. In other words, studies have shown a significant relationship between the personal and professional values; individuals who desire to help others commonly choose the catering professions [5, 6].

In clinical decision-making, the nurses reflect upon what they have learned and believe to be true; thus, these values are the foundation for nursing practice as well as the guidelines for nurses in interacting with patients, colleagues, other professions, and public [7]. These values act as the guide in performing the ethical behaviors in providing the safe care [2] and as the predictors of care quality and job satisfaction, motivation, organizational attachment, and work commitment [8]. Due to the nurses’ role in patients’ care, they are always dealing with the conditions that require them to make ethical decisions. As a result, they should be enough prepared to identify and respond to such issues [9]. In dealing with ethical dilemmas, nurses are expected to reflect the professional values in their decisions [2]. Ethical dilemmas and professional nursing values affect the quality of nurses’ professional life and play an important role in nurses’ degree of burnout and their decision upon staying in or leaving their jobs or even the profession. In the case that nursing students or nurses rely on the values that conflict with those adopted by the profession, the profession’s image and identity may be at risk [10]. Nurses’ professional life improves if they act according to the set values and beliefs in facing with the ethical dilemmas and foster establishment of the positive professional values such as freedom, human dignity, justice, and truth. Therefore, nurses should be well aware of their own beliefs, values, cultural differences, and biases in order to avoid ineffective communications in stressful situations [9]. Promotion of the professional values occurs along a continuum; it begins when a student enters the university, starts the training nursing, and continues throughout the years of nursing practice [11]. Most students and nurses tend to learn the routine clinical care skills after entering the working environment. Sometimes, they do not consider a professional value such as the patients’ refusal of treatment as an ethical issue and most of them are unaware of the patients’ rights [12].

A systematic review revealed that many nursing students and nurses reported a knowledge gap between the Code of Ethics and its application in practice [13, 14]. This knowledge gap has some potential consequences for nurses, patients, and society. Therefore, in order to develop the profession, it is essential to purposefully integrate the professional values in nursing education through value-based behaviors that guarantees the future of nursing and is rooted in the care concept [12]. Most ethical issues and values are informally and accidentally taught by unplanned curricula and clinical discussions, as a part of the curriculum. Considering the nursing educators’ roles as practical and behavioral models, they influence internalization of the professional values in students’ performance. The nursing educators play fundamental role in determining the future direction of the nurses’ professionalization and prepare them for decision-making and confronting with inevitable challenges [11]. In addition, hospitals should strengthen the professional values among the novice nurses and introduce the expert nurses to the novice nurses in order to facilitate promotion of the professional values [15]. Results of a study by Schank and Weiss showed that the professional value scores of the nurses were significantly higher than those of the senior nursing students. Another study in Spain reported that gender affected perception of ethical values among the students [4]. One study in Iran compared nurses, nursing students, and nursing instructors’ perception about nursing professional values. According to results of this study, significant differences were observed among these three groups’ perception in all these dimensions of professional values [11]. Two other studies in the USA showed no difference between the nurses and nursing students with regard to the professional values scores [10, 12]. In various studies, differences in professional values reflected differences of the communities in prioritization of the principal values based on the cultural, social, economic, and religious situations [3, 16, 17]. So, we need to be aware about importance of the professional values from viewpoints of the nurses and nursing students in different hospitals, educational backgrounds, and cultures in order to use more effective strategies for applying the professional values. Moreover, since professional values in nurses’ workplace are influenced by factors such as organizational and managerial culture and support, the professional values may change during their working period. Although most previous studies examined the professional values of students, these two groups were rarely examined and compared. Therefore, the present study was aimed to compare perspectives of the nurses and nursing students with regard to the professional values.

## Materials and methods

### Aim

The aim of this study was to compare the importance of professional values from the perspectives of nurses and nursing students.

### Study design and settings

This cross-sectional descriptive-analytical research was conducted in four teaching and referral hospitals and the large School of Nursing and Midwifery affiliated to Kerman University of Medical Sciences in southeast Iran. These hospitals have more than 1000 beds in emergency, neonate, pediatric, surgery, internal medicine, and dialysis wards as well as the coronary care, intensive care, and neonatal intensive care units to provide specialized care to patients with cardiac, endocrine, pulmonary, gastrointestinal, neurological, and psychological disorders. These hospitals receive patients from southeast of Iran, within the radius of 500 km 24 h per day, 7 days per week. Different high-tech medical and surgical interventions are conducted in these hospitals by nursing and medical staff, who collaborate with the university-based medical scientists in educating the healthcare students. Implementation of the healthcare policies is centralized in Iran and the Ministry of Health governs all the hospitals. Therefore, nurses’ conditions and distribution are similar in all hospitals around the country [18].

### Samples

The nurses population included all nurses employed in all treatment wards such as emergency, surgery, internal medicine, and intensive care units, etc. (*N* = 1113). The nursing students’ population included all undergraduate nursing students studying at the time of data collection (*N* = 177). Considering the information collected from previous studies [19, 20] and using the sample size formula) d = 0.3, 1 − β = 0/80, α = 0/05), 260 clinical nurses and 106 nursing students were invited to participate in this study. Participants were recruited by written invitation, in which they were informed about the study goals and methods. Furthermore, they were asked about their willingness to participate in the study.

The inclusion criteria for nurses included having job experience of more than one year, being directly involved in patient care, and having the bachelor’s degree and higher in nursing. For nursing students, the inclusion criteria were studying in the fourth, sixth, and eighth semesters for receiving the bachelor’s degree with no official working background in hospitals. The exclusion criteria included withdrawing from the study or handing incomplete questionnaire. The participants were selected proportionate to each hospital population in various clinical wards and each semester from the School of Nursing and Midwifery. Therefore, from the four hospitals with populations of 279, 391, 313, and 130 nurses, 64, 93, 73, and 30 nurses were enrolled, respectively. Moreover, from the three semesters with 50, 62, and 65 students, 29, 37, and 39 students were enrolled, respectively. In the nurses group, 250 individuals completed the questionnaire (response rate of 96%). In the student group, 100 participants completed the questionnaire (response rate of 95%).

### Instrument and data collection

Data collection was conducted using a two-part questionnaire. The first part collected the nurses’ demographic characteristics such as gender, marital status, level of education, income level, years of work experience, and participation in ethical training (such as code of ethics, professional ethics, professional competency). In this regard, the nursing students’ demographic characteristics included gender, marital status, educational semester, economic status of the family, grade point average, and previous participation in ethical training. The second part was Weis and Schank’s Nursing Professional Values Scale-Revised (NPVS-R). The NPVS-R is the only known instrument for measuring professional nursing values [21]. This instrument reflects the current nursing standards based on the nurses’ code of ethics with interpretive statements [22]. This questionnaire was translated into Persian and verified by Hoseini et al. [20]. A group of experts, comprising of five faculty members in Nursing and five ethics specialists, reviewed the translated questionnaire and confirmed its content validity. To ensure the reliability, a pilot study was conducted on 20 nurses and 20 nursing students, in which the Cronbach’s alpha coefficient was calculated as 0.90. The pilot study participants were excluded from the main study.

The NPVS-R consists of five dimensions: caring (9 items), activism (5 items), professionalism (4 items), trust (5 items), and justice (3 items). Participants specified the importance of each item on a 5-point Likert scale ranging from 1 to 5: not important (1 score), somewhat important (2 scores), important (3 scores), very important (4 scores), and the most important (5 scores) (see Additional file 1). The possible range of scores is between 26 and 130 [21], in which higher scores show stronger rating and orientation for the professional values.

Prior to implementation of the study, the research was approved by the Ethics Committee affiliated to Kerman University of Medical Sciences. At the study initiation stage, in order to collect the data, the first researcher provided the participants with necessary explanations about the study goals using the cover letters. Furthermore, participants were ensured about their anonymity and confidentiality of the information. Later, the nurses were randomly selected proportionate to each hospital population in various clinical wards. In the next stage, the first researcher went to the hospitals’ wards at the appropriate time and collected the data using self-reported method. Nursing students were also selected proportionate to their population in each semester using the stratified random sampling method. With regard to the nursing students studying at the fourth and sixth semesters, the data were collected at the end of one of their classes (theory course). For the students studying at the eighth semester, the required information was collected in the hospital, where they spent their internship. All data were collected using the self-reporting method and all participants were asked to put their completed questionnaires in a special box.

### Statistical analysis

Data were analyzed via SPSS version 19 using descriptive (frequency, percentage, mean, and standard deviation) and inferential statistics (independent samples t-test and ANCOVA). Pearson’s correlation coefficient and Chi-square test were also run in this regard. Chi-square test was used to compare the demographic variables of nurses and students. Independent sample t-test was applied to examine the correlation of professional values’ total score with demographic variables of gender, marriage, and participation in ethics courses. Pearson’s correlation coefficient was run to investigate correlation of the nursing values’ total score with the participants’ age. In order to control the effect of age, gender, marital status, and previous participation in ethics courses on professional values of nurses and students, the ANCOVA was used. Level of significance was set at *p* ≤ 0.05.

## Results

The results showed that the students’ mean age was 21.9 ± 1.26 years and the nurses’ mean age was 32.7 ± 7.37 years. The difference between the two groups was significant in terms of age (*p* < 0.0001). Majority of the nurses were female (90%), married (75.6%), and nearly 62.4% of them participated in the ethical training courses. Majority of the students were female (75%) and single (67%). Moreover, 16% of them participated in the ethical training courses (Table 1).

**Table 1:** Comparison of demographic information of nurses and students

variable		Nurses(*n* = 250) N (%)	Students(*n* = 100) N (%)	*P*-value
Gender	Female	225 (90%)	75 (75%)	< 0.0001
	Male	25 (10%)	25 (25%)	
Marital status	Married	189 (75.6%)	33 (33%)	< 0.0001
	Single	61 (24.4%)	67 (67%)	
Attending ethical training	Yes	156 (62.4%)	16 (16%)	< 0.0001
	No	94 (37.6%)	84 (84%)	

Table 2 shows that the perspective total mean score of nurses (3.68 ± 0.16) and nursing students (3.86 ± 0.17) toward professional values were at an important level. In addition, significant difference was observed between the nurses and nursing students regarding their total score mean in professional values. Students’ perspective on the importance of professional values was more favorable than that of nurses.

**Table 2:** Comparisons of the NPVS-R dimensions mean scores in nurses and nursing students

Dimension	Item	Nurses Mean ± SD	Students Mean ± SD	P-value
Trust	1	3.62 ± 0.18	3.82 ± 0.2	0.02
	2			
	9			
	14			
	15			
Justice	3	3.67 ± 0.21	3.86 ± 0.25	0.02
	12			
	13			
Professionalism	5	3.47 ± 0.22	3.77 ± 0.24	0.006
	6			
	7			
	8			
Activism	4	3.61 ± 0.23	3.68 ± 0.22	0.97
	10			
	11			
	19			
	26			
Caring	16 17 18 20 21 22 23 24 25	3.85 ± 0.18	3.99 ± 0.19	0.1
Total		3.68 ± 0.16	3.86 ± 0.17	0.01

The highest mean score of professional values of nurses and students was related to the dimension of care and justice, respectively. Furthermore, the lowest mean score of professional values was related to professionalism and activism in nurses and to activism and professionalism in students, respectively.

According to Table 3, the professional values’ mean scores of nurses changed significantly based on their previous participation in ethical training courses. In other words, nurses who participated in the ethical training courses obtained higher professional value scores compared to the nurses who did not participate in such courses previously. The results of Pearson’s correlation coefficient test showed a significant relationship between the NPVS-R mean scores of nurses and their age. However, no significant difference was found in professional values based on other demographic information of the participants (*p* > 0.05).

**Table 3:** Nurses and students demographic information and its relationship with NPVS-R mean scores

variable		Nurses Mean ± SD	P-value	Students Mean ± SD	P-value
Gender	Female	3.95 ± 0.45	0.07	3.96 ± 0.48	0.1
	Male	3.78 ± 0.46		3.78 ± 0.42	
Marital status	Married	3.85 ± 0.47	0.1	3.97 ± 0.48	0.39
	Single	3.96 ± 0.44		3.89 ± 0.47	
Attending ethical training	Yes	4 ± 0.44	0.007	3.8 ± 0.48	0.27
	No	3.84 ± 0.44		3.94 ± 0.47	
Age		r = 0.17	0.006	r = 0.03	0.73

## Discussion

The present study was aimed to compare the professional values from viewpoints of the nurses and nursing students. Results showed a significant difference in the mean total score of the professional values between nurses and nursing students. The professional values’ scores of the nursing students were significantly higher than nurses. These results can be justified by the fact that values learned in the nursing education programs might be changed after graduation or employment. The values change as a result of: imposition rather than free choice, lower importance of some values and lack of their sufficient approval in clinical setting, lack of motivation, work pressures, lack of organizational support for the nursing profession, organizational culture, colleagues’ values, situations experienced when caring for patients [3, 4], insufficient education on the professional values, and lack of opportunity to participate in professional associations and nursing research activities [15]. All the mentioned factors can influence the nurses’ perceptions of professional values. Moreover, it can be said that values are innate and flexible; they might change over time depending upon the everyday events that affect thought and behavior [23].

In the preset study, findings indicated that both nurses and nursing students had well-orientated professional values and internalized much of the nursing professional values. However, they did not consider each NPVS-R domain equally important, which is similar to findings of other studies [4, 11, 20, 24, 25]. It can be said that the nursing profession attracts students who inherently value (or have prior experience in) caring for and adjusting others. Furthermore, the majority of individuals in nursing profession accepted professional values as important factors in nursing, although they might not apply all of them equally in practice [26].

In our study groups, the highest mean scores of professional values were attributed to the caring domain such as “maintaining the confidentiality of patient” and “safeguarding patient’s right to privacy”. The caring items are often considered as the nurses’ most fundamental commitments and deal with concern for the patient. Justice domain was rated as the second important dimension from the perspective of nurses and nursing students. The justice domain reflected the nurses’ duty to provide equitable care to all patients such as “Assume responsibility for meeting health needs of culturally diverse population”. In this respect, studies in other countries have reported similar results [7, 8, 10, 11, 19, 27]. In these studies, the domains of care and justice or their associated values were most important from the participants’ points of view. So, it can be said that these domains are among the core values of nursing since they were selected as the most important values by the nurses and the nursing students. Moreover, these values are associated with patient care and nurse-patient relationships in clinical area, as the main focus of the ethical codes for nurses [16]. Considering the justice domain, Salminen et al. evaluated ethical principles in nursing education from the perspective of educators and students. In this study, 76% of the educators and 18% of the students mentioned justice as the main important locus of attention [28]. Consequently, Jafari et al. introduced Justice as one of the four main general principles of professional ethics [29].

In the nurse group of our study, the lowest mean scores of professional values were related to professionalism and activism domains, respectively. In our nursing students’ group, the lowest mean scores of professional values were attributed to activism and professionalism dimension, respectively. Items of the professionalism domain deal with the management and traits of a profession such as “participating in peer review” and “establishing standards as a guide for nursing practice”. In addition, activism items focus on the nurses’ role in professional organizations, public policy, and changes in health-related activities such as “participating in nursing research and/or implementing research findings appropriate to practice” and “participating in activities of professional nursing associations”. In several studies [7, 8, 10, 11, 19, 27], these domains and their items were among the most and least important values, which were consistent with the results of our research. To confirm low importance of the professionalism and activism domains in nursing professional values, it can be said that these values are related to the social and political issues in nursing profession. In this respect, Clark states that nurses mostly consider the values that are directly associated with their profession and patient care; thus, the out-of-working time activities are not much valued by nurses [16]. The motivation to participate in these activities is formed and increased after establishing professional values in the process of value training by considering all the Bloom’s learning domains, especially the affective domain, encouraging the trainers of this profession or colleagues, and having the organizational support. Students could learn to value activism and professionalism from their educators by role modeling as well as from non-faculty nurses, who are active in these values and display them obviously in their daily activities. Mutually, behavior of the nursing managers, as role models, can affect application of the professional values by nurses. Thus, the nursing managers should improve their level of awareness and skill in these values.

We found that nurse’s age had a positively significant relationship with the scores of professional values. These results are consistent with those reported by Kubsch et al. [30]. However, Al Shammari et al. indicated that no relationship was found between age and professional values [23]. It can be said that time and experience are factors that facilitate movement throughout the transformation continuum; older nurses have more experience that reinforce their professional values.

In the present study, professional value scores of the nurses who participated in the ethical training courses were higher than the nurses who did not participate in such training courses. It can be said that after graduation, assimilation of the values is affected by continuous education or participation in the workshops [2]. These findings agree with the results reported by Poorchangizi et al., who showed that the professional value scores of the nurses trained on the professional values were higher than those who did not attend training courses [26]. In a study, experienced baccalaureate students and nurses expressed the need for ethics education. This study stated that if nurses referred to the Code of Ethics as a guide rather than relying on their personal beliefs in making decision, they would have potentially saved the patient’s life [14]. Continuous education has an evident role in learning and instilling the professional values. Individuals with stronger orientations towards professional values have a higher frequency of caring behaviors [31]. Consequently, training nurses committed to ethical values are cornerstone of the decent nursing care and improve the patient’s recovery [32].

Ethical care is an integral component of the nursing profession, which entails professional training and socialization. Since the professional values are formed through professional socialization at university, it is not surprising that the paradigm of nursing and nursing education emphasize on professional values particularly. Studies showed that education caused a difference in professional values between the junior and senior students [4, 10, 33]. Since many factors such as education, attitude, and culture affect development of the professional values and ethical decisions, the educational centers should use various educational methods in order to familiarize the students with professional values according to their features [11].

One limitation of the present study was its cross-sectional nature; in which only information of a single time period was collected. However, longitudinal studies provide more detailed information on changes of the professional values over the time from the students’ entrance to the universities until they begin their work. In this vein, we recommend the future researchers to conduct qualitative or hybrid studies to obtain more accurate results. Moreover, further studies should be conducted on variables that are stronger predictors of the professional values. As another limitation of this study, the participants’ responses may be biased regarding the degree of importance they allocate to professional values, which should be considered in using the results.

### Implications for nursing education and practice

From perspective of the nurses and nursing students, some of the values were of lower importance. Therefore, it is recommended to put more emphasis on these values in educational programs of the students and clinical nurses. Results of the present study can be useful for the nursing instructors, nursing managers, and decision-makers in the field of nursing to develop appropriate strategies in promoting the nursing professional values.

These strategies may include reviewing the educational needs as well as modifying and developing the educational programs. Active research committees in universities and hospitals can promote values such as participating in studies and applying heir results. One of the strategies to create value-based behaviors is to take into consideration the role models in the universities and hospitals. It is essential to strengthen the collaboration and interaction between the universities and hospitals, because clinical situations play an important role in the process of professionalization. All of these strategies can reduce the gap between the theoretical and practical knowledge of the nurses.

## Conclusion

In the present study, the association between experience and professional value development was less clear. Although the nurses had more experience, they did not have significantly higher NPVS-R mean scores compared to the nursing students. This indicated both educational and clinical shortcomings, leading to the inadequate promotion of the professional values. Faculty members and hospital managers can develop professional values of nursing students and nurses dramatically by focusing on instilling professionalism and activism. This study suggests an opportunity for strengthening values beyond the nurse–patient relationship, such as activism and professionalism.

Nurse will need to view professionalism and activism equally important to have justice and provide care. Moreover, continuous education and managers’ support can improve the professional nurses’ ability and motivation for applying the professional values comprehensively; especially, values related to the social and political issues. Finally, the nursing education should be designed so that it can enhance the professional values and value-based behaviors. Promotion of the comprehensive professional values must be considered as an important part of the nursing profession’s socialization in the future research and educational planning.

## Supplementary information

**Additional file 1.** Nursing Professional Values Scale-Revised

## Data Availability

The datasets generated and analysed during the current study are not publicly available because this study is part of a larger study.

## Abbreviation

NPVS-R: Nursing professional values scale-revised
