# Supplementary material for: Professional Values of Nurses and Nursing Students: a comparative study
Source: BMC Med Educ. 2019 Nov 27;19:438. doi: 10.1186/s12909-019-1878-2 (PMC6882014; doi:10.1186/s12909-019-1878-2)
Supplement: Supplementary file 1 — Additional file 1. Nursing Professional Values Scale-Revised [file 12909_2019_1878_MOESM1_ESM.docx]

| most important  5 | very important  4 | important  3 | somewhat important  2 | not important  1 | Items |
| --- | --- | --- | --- | --- | --- |
|  |  |  |  |  | 1. Engage in on-going self-evaluation. |
|  |  |  |  |  | 2. Request consultation/collaboration  when unable to meet patient needs. |
|  |  |  |  |  | 3. Protect health and safety of the  public. |
|  |  |  |  |  | 4. Participate in public policy decisions  affecting distribution of resources. |
|  |  |  |  |  | 5. Participate in peer review. |
|  |  |  |  |  | 6. Establish standards as a guide for  practice. |
|  |  |  |  |  | 7. Promote and maintain standards  where planned learning activities for  students take place. |
|  |  |  |  |  | 8. Initiate actions to improve  environments of practice. |
|  |  |  |  |  | 9. Seek additional education to update  knowledge and skills. |
|  |  |  |  |  | 10. Advance the profession through  active involvement in health related  activities. |
|  |  |  |  |  | 11. Recognize role of professional  nursing associations in shaping  health care policy. |
|  |  |  |  |  | 12. Promote equitable access to nursing and health care. |
|  |  |  |  |  | 13. Assume responsibility for meeting  health needs of the culturally diverse  population. |
|  |  |  |  |  | 14. Accept responsibility and  accountability for own practice. |
|  |  |  |  |  | 15. Maintain competency in area of  practice. |
|  |  |  |  |  | 16. Protect moral and legal rights of  patients. |
|  |  |  |  |  | 17. Refuse to participate in care if in  ethical opposition to own  professional values. |
|  |  |  |  |  | 18. Act as a patient advocate. |
|  |  |  |  |  | 19. Participate in nursing research  and/or implement research findings  appropriate to practice. |
|  |  |  |  |  | 20. Provide care without prejudice to  patients of varying lifestyles. |
|  |  |  |  |  | 21. Safeguard patient’s right to privacy. |
|  |  |  |  |  | 22. Confront practitioners with  questionable or inappropriate  practice. |
|  |  |  |  |  | 23. Protect rights of participants in  research. |
|  |  |  |  |  | 24. Practice guided by principles of  fidelity and respect for person. |
|  |  |  |  |  | 25. Maintain confidentiality of patient. |
|  |  |  |  |  | 26. Participate in activities of  professional nursing associations. |
